# Aceclofenac-loaded pluronic F108/L81 mixed polymeric micelles: effect of HLB on solubilization

**DOI:** 10.1080/15685551.2022.2028373

**Published:** 2022-01-28

**Authors:** M. Senthilkumar, Sasmita Dash, R. Vigneshwari, E. Paulraj

**Affiliations:** Department of Chemistry, Annamalai University, Chidambaram, India

**Keywords:** Aceclofenac (Acl), pyrene, cloud point, pluronic surfactant, rheology

## Abstract

Pluronic block copolymers have phase behavioural characteristics which are extensively studied for drug delivery applications. In this study, we explored hydrophilic pluronic F108 (HLB = 27), hydrophobic pluronic L81 (HLB = 2) and their mixed micelles acting as solubilising mediums for model drug aceclofenac. The drug solubilisation and interactions have been analysed using UV-visible spectroscopy, Fluorescence spectroscopy, Rheology studies, Fourier-transform infrared spectroscopy, Scanning electron microscope, Dynamic light scattering, Cloud point and partition coefficient measurements. The investigation from UV-spectrophotometry demonstrated that mixed pluronic entrapped greater number of aceclofenac molecules than both the neat pluronics at same concentration. Excimer formation was evidenced from fluorescence spectra with pyrene as a probe. The rheological studies showed difference in viscosity over low shear range. Studies on FTIR demonstrated probable bonding between the aceclofenac and mixed pluronic molecules. The DLS studies on mixed pluronic showed swelling of micellar diameter from 317.6 nm to 413.5 nm. Thermodynamic parameters of the above system revealed higher partition coefficient value for mixed pluronic and spontaneity in drug solubilisation. This study can be exploited to use a hydrophobic copolymeric micelle in mixed pluronic formulation for better drug solubilisation.

## 1. Introduction

Commercially available pluronic block copolymers encompass ethylene oxide (EO) and propylene oxide (PO) blocks present structurally as EOxPOyEOx. Their molecular characteristics are available over a wide range, with varied PO/EO ratio. Because of their amphiphilic and micelle-forming properties, this group attracts researchers from various specialities for applying them in solubilisation and delivery of drugs which are poorly soluble [1–4]. Because it contains both hydrophobic and hydrophilic moieties in the same molecule, the pluronic group has numerous advantages over conventional surfactants. When the molecular weight is low, the pluronic remains in the form of pastes and viscous oils, and when high, it exists as amorphous solids. The varieties of blocks present inside the copolymer are incompatible among themselves and due to that block copolymers self-congregate inside solutions and melts. They display anomalous behaviour in a particular temperature-range in individual solvent medium. The range of concentration interval for CMC of pluronics is much larger than that of conventional surfactants. Single pluronic micelles dominated research on delivery of drugs until recent years. Of late, the binary systems comprising of two pluronic copolymers have got more attention due to their advantages. Certain aspects of a single micellar system such as larger particle size, low drug loading ability and low stability have undergone improvement in a system of mixed micelles [5]. For example, a system of mixed micelles of pluronic L81/P123 developed by Mourya et al. showed to be containing smaller sized particles with greater solubilisation potential [6]. A system of mixed micelles of pluronic P123 and F127 was utilized in successful loading of paclitaxel (PTX) with increased antitumor efficacy [7–9]. Doxorubin loaded in a system of mixed micelles comprising of pluronics L61 and F127 was the first mixed micellar formulation that was used in the cancer chemotherapy [10]. The binary system comprising of pluronic L101 and P105 was used for incorporation of paclitaxel (PTX) for use in multidrug resistance tumors [11]. Mixed micelle containing several hydrophilic (F68, F87, F127, P85 and P105) and hydrophobic (L61, L81, L101 and L121) pluronics have been prepared and characterised [12]. Out of many tried combinations, the mixture of L121 and F127 most effectively produced small size particles, stable dispersions and high solubilisation of the Sudan-III dye in comparison with F127 single micelles. But, none of the known systems of mixed micelles has successfully engendered particles with small sizes which is desirable for the pharmaceutical formulations [10,13]. Hence, this work focuses on attempting to produce a system of mixed micelles which is stable, optically transparent by mixing F108, a hydrophilic and L81, a hydrophobic pluronic; and using the system to confer higher loading capacity on the drug pluronic F108, although a hydrophilic copolymer is not commonly used to solubilise hydrophobic drugs, probably due to its high HLB value 27. We have created a binary system comprising hydrophobic pluronic L81 and hydrophilic pluronic F108 surfactants in different ratios. The model drug aceclofenac was chosen as the hydrophobic drug whose interaction with mixed and single pluronics was compared. A similar study on mixed pluronic micellar system (pluronic L81 and P123) with aceclofenac has been reported where the HLB was L81-2 and P123-8. It was therefore of interest to observe the aceclofenac solubility in a binary pluronics system of very low and very high HLB, HLB = 2 (L81) and HLB = 27 (F108) to assess to role of HLB on the solubility and other properties.

**Table 1.** Average size and polydispersity index of aceclofenac and pluronic in aqueous solution measured by dynamic light scattering

Pluronic	Z-Average (d.nm)	PdI
L81	222	0.376
F108	17.09	0.08
L81+ F108 mixture	317.6	0.334
F108+ aceclofenac	380	0.355
L81+ aceclofenac	360.4	0.377
L81+ F108 mixture+aceclofenac	413.5	0.36
L81+ F108 mixture+ aceclofenac+NaCl	375.7	0.399

## 2. Materials and methods

### 2.1. Materials

Samples of pluronic (Sigma Aldrich) F108 and L81 with a molecular weight of 14,600 and 2750 g mol^−1^, respectively, were used with no further purification. Aceclofenac was procured from MMC Healthcare Ltd. Dimethylsulphoxide (DMSO) and Pyrene samples (Sigma Aldrich) were procured. All the reagents were of analytical grade. Triply distilled, water was used for the experiment.

#### 2.1.1. Preparation of mixed micellar systems

5% w/v stock solutions of both pluronics F108 and L81 in distilled water were prepared and then refrigerated for 48 h at 5°C. For the preparation of mixed micelle, 10 ml each of 5% F108 and L81 were mixed and, then, stored at 5°C for 12 h. These samples were kept at room temperature for stabilization before the characterization, in order to allow complete formation of aggregated structures. 2 M solution of sodium chloride was prepared.

#### 2.1.2. Preparation of drug sample

Preparation of aceclofenac in aqueous solution was made through solvent evaporation method using DMSO solvent [2]. Drug solutions, in different concentrations, were mixed with F108, L81 and mixed pluronic solutions. They were allowed to stabilize for 2 h at room temperature prior to analysis. Concentrations of the copolymer solutions were chosen well above the CMCs of F108 and L81; so that the aggregated structure could be ensured in mixed micellar solution. We have used the term ‘mixed pluronic‘ for the above composition throughout results and discussion.

### 2.2. Characterization of mixed micelles

#### 2.2.1. UV spectrophotometry

Spectral measurements of the aceclofenac and pluronic samples in different combinations were conducted using Shimadzu UV-2600 PC spectrophotometer with 1 cm quartz cell. Stock solution of Acl 1000 µg ml^−1^ was prepared by dissolving 0.1 g of pure aceclofenac in 10 ml dimethylsulphoxide (DMSO). Then it was made up to 100 ml by adding distilled water. The solutions of 5% pluronic F108, 3% L81 and mixed pluronic were prepared in 1000 µg ml^−1^ Acl; and their UV- spectrophotometric measurements were recorded.

#### 2.2.2. Fourier transform infrared spectroscopy

The Fourier transform infrared spectroscopy of Acl alone and with mixed pluronic at 298 K was noted in the range of 700 to 2500 cm^−1^ by using Cary-630 FT-IR Agilent Technology. 2 ml solution of mixed pluronic, aceclofenac and combination of both were taken and their individual spectrums were recorded.

#### 2.2.3. Fluorescence spectroscopic measurement

5 ml of 10^−6^ M Pyrene solution, as fluorescent probe, was added to the three micellar solutions, viz. L81, F108 and mixed pluronic and fluorescence measurements were taken in a LS-55 Perkin Elmer. Pyrene spectrums were recorded at excitation wavelength of 335 nm. The emission spectrums were recorded over the range of 335–600 nm. The emission wavelength was fixed at 393 nm.

#### 2.2.4. Rheology characterization

Measurements for rheology studies were made using MCR 301 Rheometer (Anton Paar, Germany – double gap cylindrical geometry) in strain-controlled mode with cone plate geometry (diameter 50 mm, angle 1°). These measurements were done at 25°C.

#### 2.2.5. Scanning electron microscope (SEM)

To study the morphological and topographical aspects of the surface of the sample, SEM was performed using JSM-5610 LV instrument JSM-5610 LV which allowed observation of sample up to 32 mm diameter. With a high resolution of 3.0 nm at 30 kV, it delivered clarity of the finest structures. The SEM images of mixed pluronic and aceclofenac encapsulated mixed pluronic were recorded.

#### 2.2.6. Dynamic light scattering measurements

The DLS studies were conducted using Malvern zeta sizer Nano-ZS instrument at 25°C ± 0.1°C. The light source used for the study was 4 mW He-Ne laser (633 nm) and the scattering angle was 173°C for all the aliquots.

#### 2.2.7. Partition coefficient (P)

P is the ratio of the drug concentration in water and pluronics at equilibrium. This was determined through the equation

P=Cm/Cw

Cm and Cw being the drug concentration in micelle and water, respectively.

The standard free energy associated with micellization was determined by the equation

$$(\Delta {G^{o}}_{mic})=-RT\left[lnP\right]$$

Here, R is the universal gas constant and T is the absolute temperature.

#### 2.2.8. Cloud point

Cloud points were determined for neat 2.0% pluronic L81, mixed micelle of L81 and F108. For neat F108, the cloud point could not be detected because it is close to 100°C and not possible in the present experimental condition. The next set of aliquots was prepared with drug aceclofenac. In the last step, the binary solution with drug and salt NaCl was prepared and cloud points were measured. The first appearance/disappearance of turbidity was assumed to be the cloud point.

## 3. Result and discussion

### 3.1. UV-visible spectroscopy

**Figure 1.**
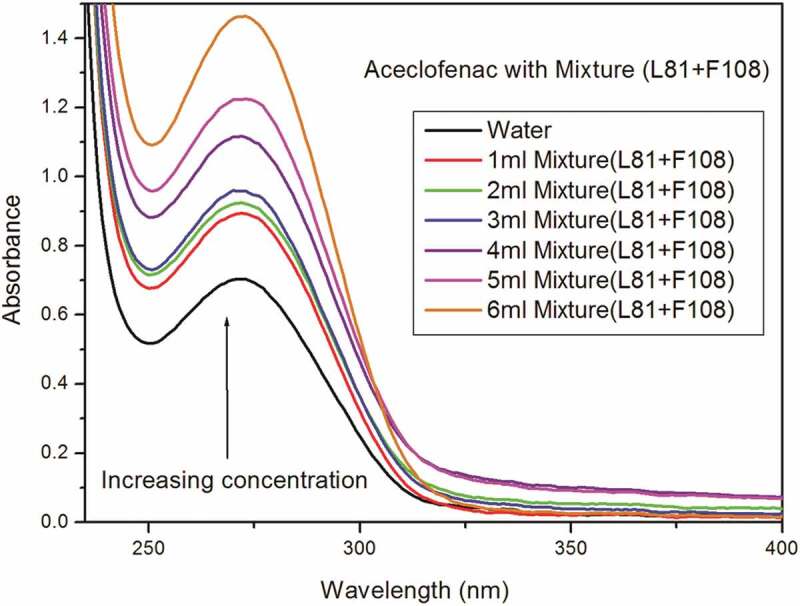
Absorption spectra of multiple concentrations of mixed pluronic with aceclofenac.

**Figure 2.**
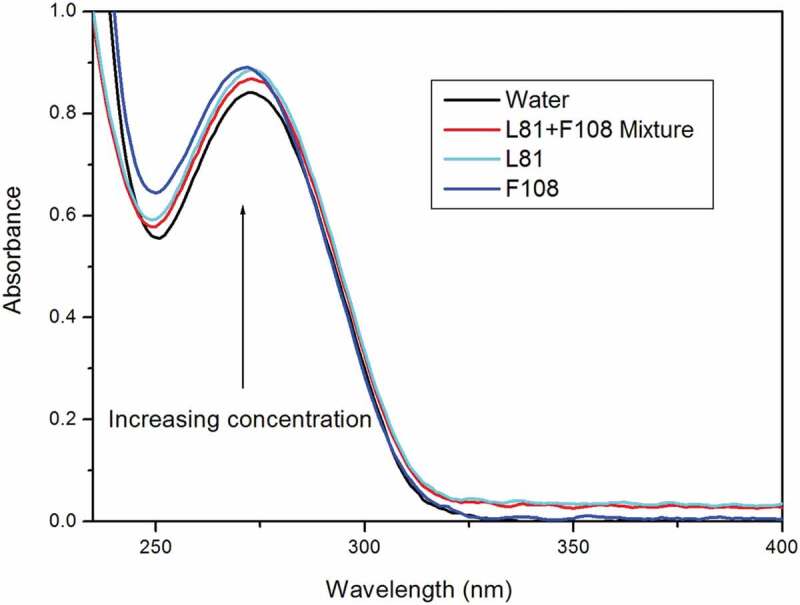
UV-absorption spectra of aceclofenac with single pluronic and mixed pluronic.

UV-visible spectra is used to know the hydrophobicity and complex formation occurring in drug-surfactant interaction. The aqueous drug solution of Acl showed the maxima at 274 nm [6,14–16]. The structures of drug aceclofenac, pluronic L81 and pluronic F108 are included in supplementary sections Figure S1, S2 and S3. On adding neat or mixed pluronic solution to the drug, no shift of wavelength was noted. In this study, the mixed pluronic (F108+ L81) was prepared in four different combinations, viz. 30% + 70%, 50% + 50%, 70% + 30% and 80% + 20% to identify the most suitable one for solubility of the drug aceclofenac. The pluronics F108 and L81 were selected based on their hydrophobicity, HLB and molecular weights. The UV spectra of aceclofenac alone in aqueous medium was taken as blank. In the following step, fixed volume of mixture of 70% F108 and 30% L81 was added. This chosen combination displayed maximum absorbance out of the four combinations. The absorbance of mixed-pluronic drug lay in between the two single-pluronic-drugs (Figure 2). The spectra (Figure 1) reveals that the absorption continuously increases with rise in concentration of mixed-pluronic solution. This proves that the number of molecules of the drug aceclofenac, entrapped in the mixed micellar system, is greater at higher concentrations (Figure 2.) The enhanced solubility of Acl in the mixed-pluronic system at higher concentration can be assigned as stronger interaction between drug and mixed pluronic. This finding is in consonance with reports of other researchers [9,17]. They have described that the CMC of the system of mixed micelles lay in the middle of that of the single micelles. The hyrdrophobic pluronic L81 has potential to solubilise poorly water soluble drugs. But, the drug delivery is not very efficient because of the large, aggregated lamellar structures and their instability in aqueous medium. In a mixed pluronic system, however, the two block copolymers generate the micelles through hydrophobic interactions of both the PO units of copolymers. This helps the L81 to give a clear micellar dispersion in aqueous medium. In the present study, the combination of low and high HLB led to a system to accommodate more number of drug than the single micelle of hydrophobic L81. The increase in absorbance of single pluronic was specific to drug-pluronic combination; and it reflects the degree of drug-pluronic interaction. Pluronic F108 displayed absorbance value higher than L81 which is, probably, due to hydrophilic nature of the former.

### 3.2. Fluorescence spectroscopic measurement

**Figure 3.**
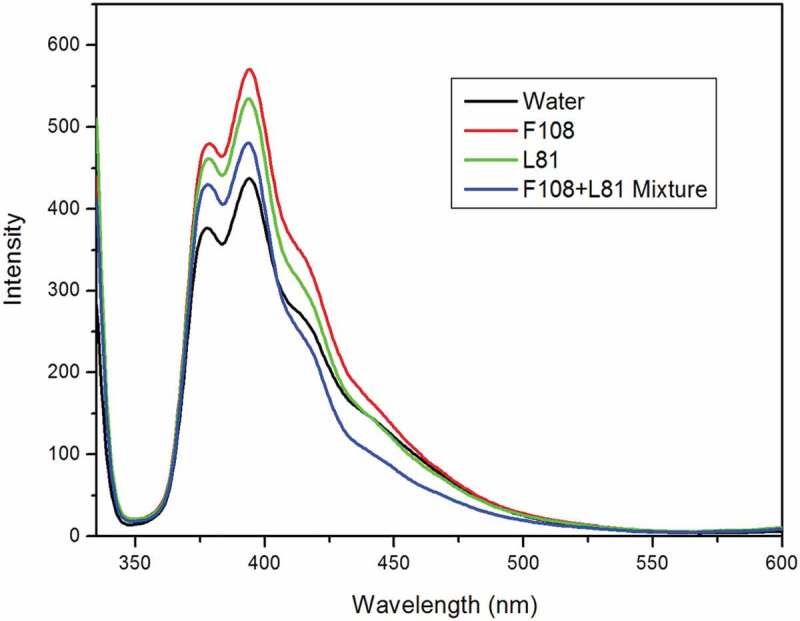
Fluorescence spectra of aceclofenac with single pluronic and mixed pluronic system in presence of pyrene.

**Figure 4.**
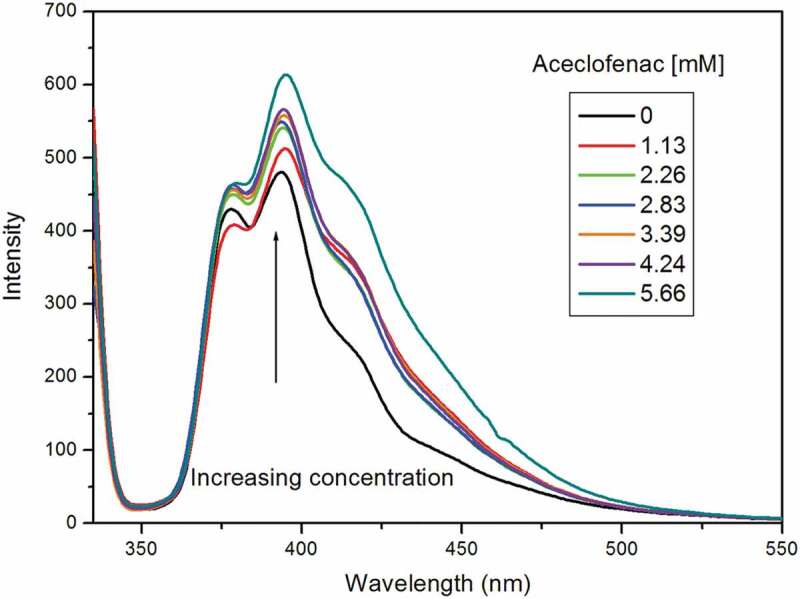
Fluorescence spectra of aceclofenac (different concentrations) with mixed pluronic system in presence of pyrene.

Fluorescence excitation and emission spectrum of pyrene in the presence of the drug aceclofenac, neat pluronic and pluronic mixtures are displayed in Figure 3. Pyrene monomer, in aqueous solution, demonstrated the classic vibronic structure at 384 nm [18,19]. Addition of pluronic F108 did not change the configuration of spectra excepting that there was increase in intensity. With addition of L81, similar result was obtained excepting that the rise in intensity was less than the former (Figure 4). The intensity in the spectra is created by the microenvironment generated by the pluronics. The structure as well as polarity of the drug determines its position in the micelle. On adding pluronic mixture to the pyrene, there was reduction in intensity compared to both the neat pluronic spectra. There was no change in the vibronic structure. In the following step, drug aceclofenac was added to mixed pluronic in pyrene. In addition of increase in concentration of drug, there was continuous enhancement of intensity [20–28]. This indicates encapsulation of drug in the micellar hydrophobic core. There was no shift of the spectra and the spectral characteristics were seen to be the same on drug addition. The possible explanation for the above observation would be excimer formation because of the combination of pyrene and drug aceclofenac with pluronics. The excimer formation suggests that pyrene molecule exists in two types of environments (i) isolated monomers (at low drug concentration) and (ii) excimer (at high drug concentration). In the second case, the encapsulated drug molecule and the probe are in proximity; hence, there may be interaction between them even when present in ground state forming a complex. Similar findings have been earlier reported in literature [29].

### 3.3. Rheological studies

**Figure 5.**
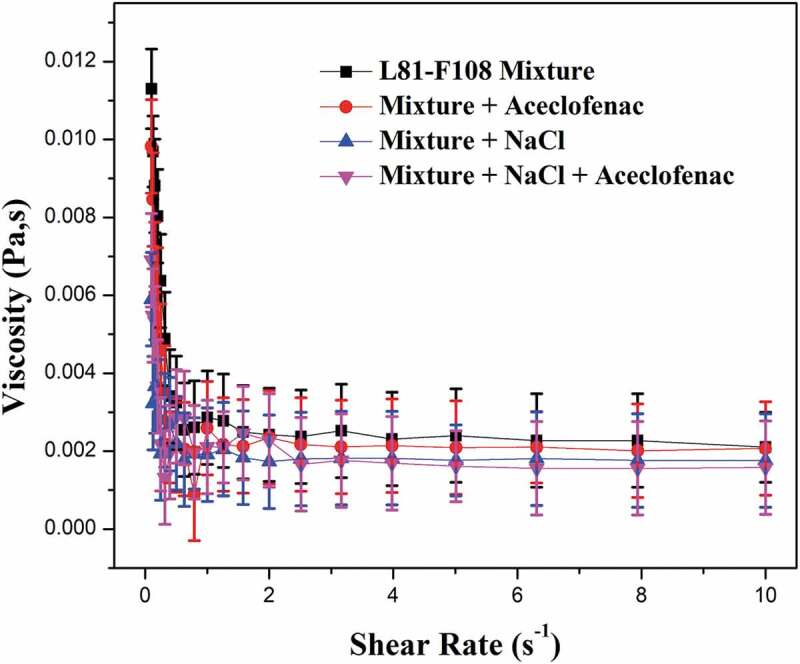
Rheological behaviour of pluronic (5%) L81+ F108 mixed pluronic, mixed pluronic with aceclofenac, Mixed pluronic with NaCl, and Mixed pluronic with NaCl and aceclofenac.

Viscosity measurements at different shear rates for mixed pluronic, mixed pluronic–drug aceclofenac, mixed pluronic–sodium chloride, mixed pluronic–drug aceclofenac–sodium chloride were carried out. They are displayed in Figure 5. The mixed pluronic–drug aceclofenac combination showed to have lower viscosity compared to mixed pluronic alone. This lowering was observed only when shear rate was low. The reason for this may be due to the bonding that has been formed on encapsulation of drug aceclofenac with mixed pluronic micelles. Beyond a paricular shear rate (1 s^−1^), the viscosity was almost constant for both the samples described above. Out of four combinations studied here, the viscosity reduction is maximum for mixed pluronic–drug–salt combination. The lowering of viscosity could be due to the extra bonding incorporated in the system making it bulkier. For every system, there is existing a balance involving all the forces (interactions) among the micelle, drug and or other substances (like sodium chloride here). Factors such as micellar size, concentration of solution and lifetime on the interaction also has a role to play. When the shear rates are low, the differences in the viscosity are more significant as observed by many researchers [30–32]. When shear rate increases, the hydrodynamic participation dominates and because of that the shear viscosity curves approach each other.

### 3.4. Fourier transform infrared spectroscopy (FT-IR)

**Figure 6.**
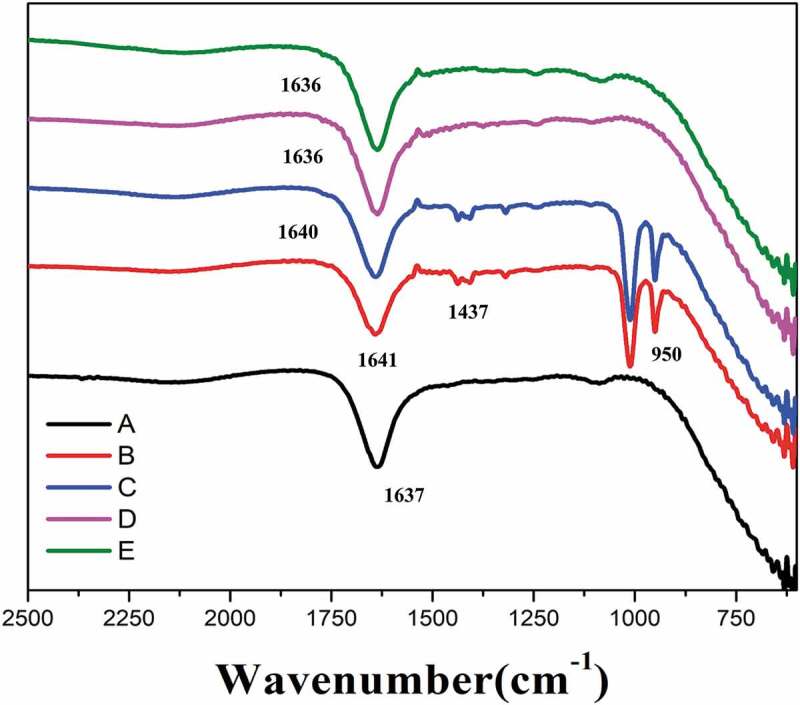
FT-IR spectra of (a) L81+ F108 mixed pluronic, (b) aceclofenac, (C) aceclofenac with mixed pluronic, (d) pluronic L81 and (e) pluronic F108.

Fourier transform infrared spectroscopy studies are helpful in identifying different functional groups [29,33–36]. The interaction of mixed pluronic–aceclofenac was visualized from the IR spectra between 700 and 2500 cm^−1^. The peaks of FTIR are shown in Figure 6(d,e) which display the IR spectra of neat pluronic L81 and pluronic F108, respectively. Figure 6(a) shows that of the pluronic mixture. Figure 6(b,c) displays the spectra of neat aceclofenac–pluronic mixture, respectively. The spectra of the F108, L81 and F108 + L81 mixture were almost similar. Now, comparing the IR spectra of Figure 6(b), aceclofenac and mixed pluronic–aceclofenac in Figure 6(c), one can see that the C–O stretching vibration at 1011 cm^−1^, C=O vibration at 1641 cm^−1^ and O–H bond at 950 cm^−1^ remain unchanged after combining the mixed pluronic and drug aceclofenac. There is shift of C–C stretching due to aromatic ring from 1437 cm^−1^ to 1407 cm^−1^ for the same set (i.e., drug aceeclofenac (B) to drug aceclofenac + mixed pluronic (C)). Here, the probable reason can be that the shorter and strong bond in aceclofenac (B) becomes longer and weaker after combining with mixed pluronic (C). In the process, the light atom of neat drug molecule aceclofenac with high energy becomes heavier due to complex formation resulting in a product which has lower energy leading to stability of the complex. Hence, it can be inferred that drug Acl is more stable in the mixed pluronic medium.

### 3.5. Scanning electron microscopic observation (SEM)

**Figure 7.**
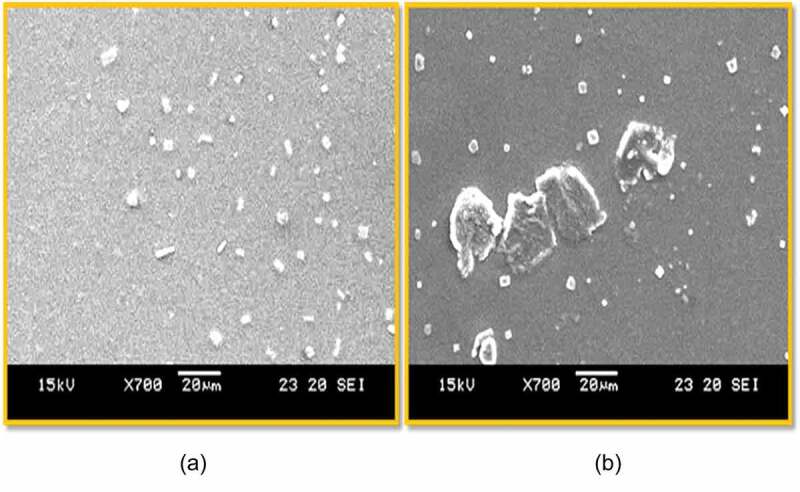
SEM image of (a) mixed pluronic (L81+ F108) and (b) aceclofenac encapsulated mixed pluronic.

Scanning electron microscopic technique was employed to visualize the surface of morphology mixed pluronic and the aceclofenac encapsulated mixed pluronic. The films of mixed pluronic on addition of drug aceclofenac was observed to change. The images of SEM are dispayed in Figure 7(a,b). As observed, the mixed micellar surface was somewhat uniform (Figure 7(a). In Figure 7(b), however, there was irregular shape, the surface morphology looks like bigger flakes appearing as patches on the uniform surface background of the mixed micelle. Pluronics micelles are known to display such SEM images [37–40].

### 3.6. Dynamic light scattering measurements

**Figure 8.**
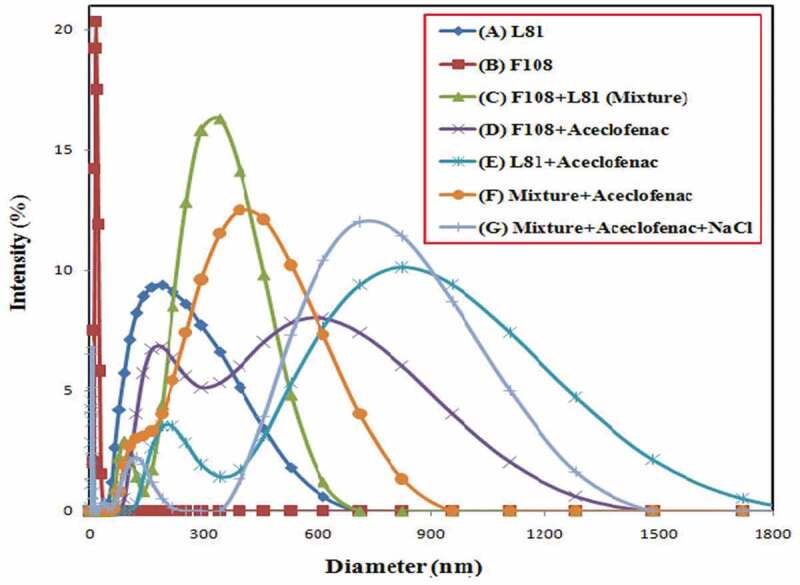
Dynamic light scattering data of 2.5% pluronic F108, L81 and mixed pluronic at 25°C.

The drug encapsulation leads to structural changes in the micelles. These changes are observed by DLS measurements. In the first step, 2.5% pluronic F108, L81 and mixed pluronic solutions were taken for dynamic light scattering study. Following this fixed concentration of drug was mixed to each of the above aliquots and particle size were measured. Finally sodium chloride 0.5 M was added to mixed pluronic, drug and change in size of the particle was observed. The results have been presented in Table 1 and Figure 8. The micellar size was seen to have diameter 222.0 nm, 17.0 nm and 317.6 nm for neat pluronic L81, neat pluronic F108 and mixed pluronic, respectively. The drug addition to all the three samples changed the diameter to 360 nm, 380 nm and 413 nm respectively. There is maximum increase in diameter of 363 nm for F108 because of hydrophilic nature of surfactant. The hydrophobic pluronic L81 (HLB-2) dominated its property in mixed pluronic in the change of size. There are dynamic light scattering reports on pluronic surfactant [41–48]. Sodium chloride addition, however, showed shrinking in micellar size to 375.7 nm from 413.5 nm which infers that there is no enhancement of drug encapsulation. The probable reason could be generation of aggregates exhibiting micelle-micelle interaction due to lack of water molecules surrounding corona. As a consequence, there may be delay and incomplete micellization occurring for mixed pluronic, aceclofenac and sodium chloride combination. Hence, there is size reduction of the micelle. Analogous findings have been reported [49–52].

### 3.7. Partition coefficient

The solubilization of drug depends on the solubilising medium, i.e., the nature of copolymeric surfactant, its hydrophobicity, its molecular weight etc. Table 2 displays the data of partition coefficients and the Gibb’s free energy of micellization in three systems viz. hydrophilic pluronic F108–water, hydrophobic pluronic L81–water, and mixed pluronic–water [41,53]. As can be noticed, the value of partition coefficient is 1.14 for the pluronic mixture which is higher than that for neat pluronic F108 (1.13) or neat pluronic L81 (0.64). This reveals that the quantity of drug solubilised in mixed pluronic is more compared to the neat pluronic ones. Although the hydrophobic L81 with (HLB – 2) was used for mixed pluronic with hydrophilic F108 (HLB – 27), the free energy data are encouraging. The (ΔG^o^_mic_) values were calculated from the partition coefficient using expression mentioned in 2.2.7. The (ΔG^o^_mic_) values for pluronic F108, pluronic L81 and mixed pluronic are −316.84, 1097.05 and −336. 76 kJ mol^−1^, respectively. The negative (ΔG^o^_mic_) indicates that there is spontaneous inclusion of drug in the mixed pluronic solutions. The higher negative value of (ΔG^o^_mic_) for mixed pluronic concludes that there is favoured solubilisation of aceclofenac drug because of stronger hydrophobic interaction between micellar core of mixed pluronic-drug compared with that of pluronic L81-drug. Normally hydrophobic copolymers are not used for drug solubility. But, it is possible to make use of a hydrophobic copolymer L81 to solubilise the drug aceclofenac by using it in a mixed pluronic form. This corroborates with the UV visible absorption studies.

**Table 2.** The partition coefficient and free energy of micellization of drug aceclofenac with single and mixed pluronic aqueous solutions

S. no	Pluronic	Partition coefficient (P)	(∆G^o^_S_) kJ. mol^−1^
1	F108	1.13	−316.84
2	L81	0.64	1097.05
3	Mixed pluronic (F108+ L81)	1.14	−336.76

### 3.8. Cloud point determination

The temperature at which the pluronic surfactants show turbidity is called cloud point. The knowledge of cloud point is important to determine storage stability. The change in the temperature for various mixtures of polymeric surfactant with and without drug aceclofenac were measured. The results are displayed in Table 3. The cloud point for 2.0% pluronic L81 was observed to be 20°C. The value is in good agreement with literature [54]. For this, an ice cube bath was prepared and temperature from 5°C onwards were noted. The craft point or cloud point of the binary mixture was observed to be 24°C. Addition of drug aceclofenac to pure L81 and the binary mixture showed the cloud point to be 18°C and 21°C, respectively. A lowering of the cloud point on addition of drug (from 20° C to 18° C and from 24° C to 21° C) predicts that the drug helps to dehydrate the copolymer micelles in both single as well as binary systems. In the above mixture, there are more number of L81 molecules in solution than F108. This is considered taking account of the molecular weight of both copolymers (L81 = 2750 g mol^−1^ and F108 = 14,600 g mol^−1^). Hence, the drug aceclofenac is having more interaction with L81 which has greater hydrophobicity than the binary micellar combination. Due to the presence of more number of PEO moiety on F108, the building up of additives in the micellar zone becomes difficult. The change in concentration of F108 in the pluronic mixture did not affect the cloud point substantially. It is presented in fourth and fifth aliquots in Table 3. Addition of salt sodium chloride increased the cloud point by 2° C (from 24° C to 26° C). This may be due to change in the conformation of PEO arising for addition of salt.

**Table 3.** Cloud point of pluronic L81, L81+ F108 with and without drug aceclofenac

S.no	Sample details	T ^o^ (C)
1	2 ml L81 + 3 ml water	20
2	2 ml L81 + 1 ml aceclofenac+2 ml water	18
3	2 ml L81 + 2 ml F108 + 1 ml water	24
4	2 ml L81 + 2 ml F108 + 1 ml aceclofenac	21
5	2 ml L81 + 0.2 ml F108 + 2 ml aAceclofenac	21
6	2 ml L81 + 2 ml F108 + 1 ml NaCl	26

## 4. Conclusion

Pluronic L81 has been reported to have very low HLB (2). It is explained by its EO: PO ratio. This polymer has not been used widely for drug solubility due to its hydrophobic nature. This paper presents the analysis of interaction of drug Aceclofenac (Acl) with mixed pluronic copolymer of F108 and L81 using several methods. The UV studies showed that more drug could be accommodated in the host micelle of mixed pluronic than the single micelle of hydrophobic L81. The fluorescence measurements displayed enhancement of fluorescence intensity because of excimer formation. The rheological studies showed that mixed micelle undergoes lowering of viscosity when drug and sodium chloride are added. The FTIR studies suggested formation of a mixed pluronic-drug complex; and that the complex possesses lower energy and greater stability than the drug alone. The DLS studies displayed that there is swelling of the mixed pluronic micelle due to incorporation of drug (Aceclofenac). The partition coefficient studies suggest that there is spontaneous and stable binding of the drug (Aceclofenac) with mixed pluronic having (ΔG^o^_mic_) value −336.76 kJ mol^−1^ compared to pluronic L81 value 1097.05 kJ mol^−1^. The attempt of using the hydrophobic copolymer pluronic L81 with very low HLB has been accomplished to solubilize aceclofenac in a mixed pluronic combination.

## Supplementary Material

Supplemental Material
