# Supplementary material for: Aceclofenac-loaded pluronic F108/L81 mixed polymeric micelles: effect of HLB on solubilization
Source: Des Monomers Polym. 2022 Jan 28;25(1):1–11. doi: 10.1080/15685551.2022.2028373 (PMC8803101; doi:10.1080/15685551.2022.2028373)
Supplement: Supplemental Material [file TDMP_A_2028373_SM7408.doc]

**SUPPLEMENTARY MATERIALS**

**Aceclofenac-loaded Pluronic F108/L81 mixed polymeric micelles: Effect of HLB on solubilization**

M. Senthilkumar, Sasmita Dash*, R. Vigneshwari and E. Paulraj

Department of Chemistry, Annamalai University, Chidambaram-608 002, Tamilnadu, India

* Corresponding author. E-mail address: [mishra342sas@gmail.com](mailto:mishra342sas@gmail.com)

***Corresponding author**

Dr. Sasmita dash

Professor

Department of Chemistry

Annamalai University

Annamalai nagar-608002

Chidambaram

Tamilnadu, India.

E-mail: [mishra342sas@gmail.com](mailto:mishra342sas@gmail.com)


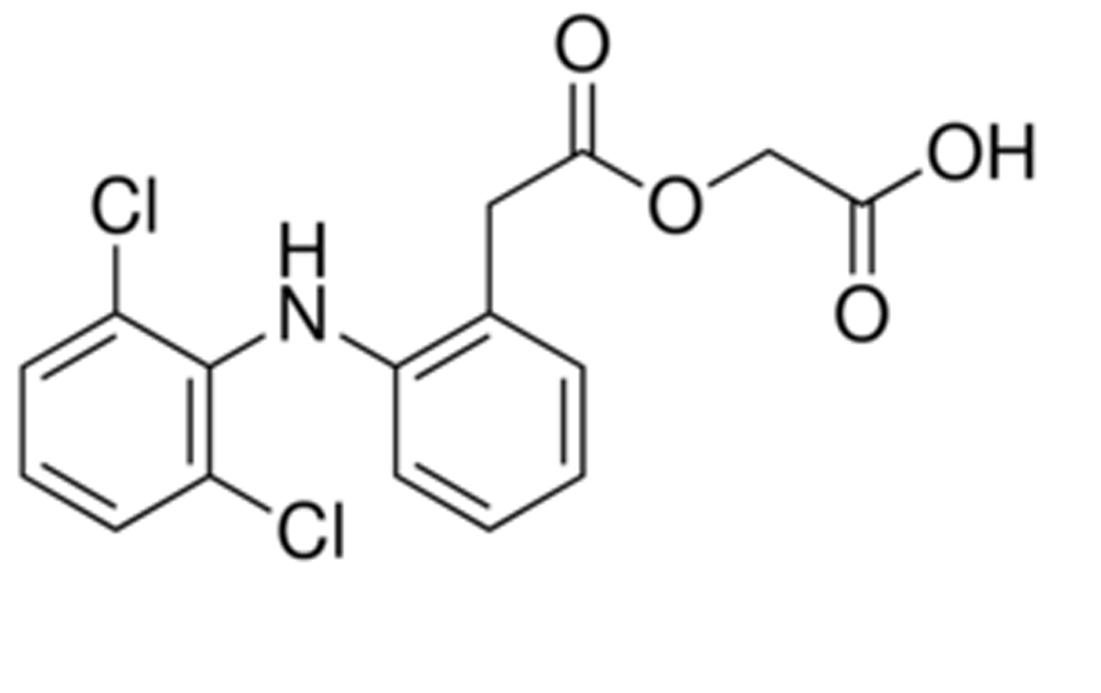


Figure S1. Structure of Aceclofenac.


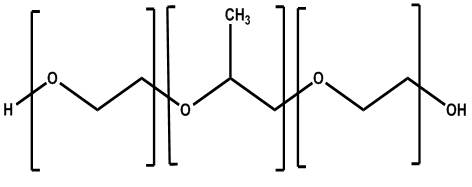


**2**

**4**

**6**

Figure S2. Structure of Pluronic L81.

Figure. S3. Structure of Pluronic F108.


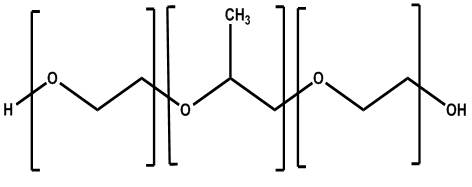


**132**

**50**

**132**
